# Clinical Lectures on Diseases of the Eye

**Published:** 1865-11

**Authors:** E. L. Holmes

**Affiliations:** Chicago, Lecturer on Diseases of the Eye and Ear in Rush Medical College, and Surgeon to the Chicago Charitable Eye and Ear Infirmary


### CLINICAL LECTURES ON DISEASES OF THE EYE.

By E. L. HOLMES, M. D., of Chicago,

*Lecturer on Diseases of the Eye and Ear in Rush Medical College, and Surgeon to the Chicago Charitable Eye and Ear Infirmary.*

### CATARACT.

GENTLEMEN:

Cataract is an abnormal change in the form and character of the cell elements of the lens, producing opacity. Its chief symptoms are, the presence of a cloudy appearance either in or behind the pupil, and a greater or less indistinctness of vision.

You will find no difficulty in the diagnosis of this disease, if you have formed the habit of examining carefully the eyes of different individuals in health, observing the appearance of the surface of the cornea and the condition of the pupil.

In the incipient stages of cataract, where there may be doubt, you can easily remove the doubt by dilating the pupil with atrophia, and throwing into it, by means of a concave reflector or a double convex lens, the concentrated light from a window, or better, the light of a candle in a darkened room. The strong light thus thrown upon any opaque substance that may exist behind or in the pupil, will so illuminate it, that it can readily be discovered on looking obliquely from different directions into the eye. In the absence of a mirror or lens, a small piece of looking glass may be used as just described, with advantage in the examination of cataract.

A little care will prevent you from confounding cataract with opacity of the cornea and save you the mortification of sending patients, with the latter disease, to some distant oculist for the removal of cataract, as has not unfrequently been done even by physicians of long experience.

A few words will suffice to explain the important points in the pathology of cataract. The lens, from abnormal nutrition, receives or loses undue proportions of certain elements. The solid or fluid materials may be in excess. The cells may contain too much fat or too much lime. Water may be absorbed in such quantities into the lens, as to disarrange and soften its cell structure. With fat globules there may be minute crystals of cholesterine, presenting a very beautiful appearance under the microscope. A peculiar form of "fatty degeneration" seems to occur as the principal feature in nearly all cataracts.

It should be remembered that the capsule resists to a remarkable degree all tendencies of abnormal conditions of the eye to render its tissue opaque. While the substance itself of both the cortical portion and kernel of the lens may be so changed in character, that they become opaque, the capsule, except in rare instances, retains its perfect normal structure, any apparent opacity being caused by the deposit of cells upon its surface. A comparison in this respect might be made between the capsule and lens, in abnormal conditions of the eye, producing cataract, and a thin plate of fine glass becoming apparently opaque on exposure to the influences of moisture and dust.

Iritis and penetrating ulcers of the cornea often produce a deposit of opaque lymph upon the anterior surface of the capsule. Chronic choroiditis is very liable to so disturb the nutrition of the lens as to render it opaque. Concussions often produce cataract. In such cases the cataract often seems to depend upon a rupture to a greater or less extent of the zonula of zinn.

Punctures of the lens are a frequent cause of cataract by permitting an undue imbibition of aqueous humor.

The causes of idiopathic cataract are very obscure. Advanced age and infancy seem especially liable to it. While I have generally found aged cataract patients in good health, I have observed that infants with congenital cataract almost always present signs of arrested development. Diabetes and long use of rye bread impregnated with ergot, are said to induce cataract. It is sometimes hereditary.

The disease may extend through the whole substance of the lens and over the whole surface of the capsule, or it may be confined to a very small central kernel; to one delicate lamina of the lens, or even to a small disk in one of its layers.

Cataracts are usually classified as cortical, central or capsular, referring to the portion affected—and hard or soft, according to the degree of induration.

You have had opportunities of examining nearly every form of cataract at the clinic.

By calling to mind these cases you will now better comprehend their nature. In the case of the young child with congenital cataract, the lens really resembled a transparent sack filled with milk. On puncturing the lens with the needle, the anterior chamber became filled with white fluid, in which was floating minute cloudy flakes. The lens had, as it were, become fluid. In the boy from whom I extracted the two cataracts through linear incisions in the corneæ, the whole of each lens was slightly opaque, with a small central portion almost perfectly white, both portions being soft.

In the case of the old lady, whom a few of you saw recently, the cataract was peculiar. You remember that when the pupil was dilated, the whole pupillary disk presented an almost uniform thick cloudy appearance. Whenever the patient turned her head from one side to the other, a small yellow body was observed to be in different positions against the anterior part of the capsule. As the cataract passed through the cornea with the capsule unruptured, it was found that the capsule was filled with a thin milky fluid, surrounding a small but very hard central kernel of the lens.

In one case the cataract had become liquefied, and finally absorbed, leaving the anterior and posterior halves of the capsule floating in close union with each other behind the pupil.

Less frequently in fluid cataracts the oil globules are found deposited in such quantities, that they unite in one large drop and float upon the other fluid.

The so-called black cataract is produced by the exudation of blood in the anterior chamber, the coloring matter of which becomes infiltrated through the capsule into the superficial layers of the lens.

Calcareous deposits in the lens sometimes depend upon chronic inflammation of the choroid and iris, as seemed to be the case in the boy, upon whom I performed Iridectomy with extraction of the peculiar chalky cataract.

From these and other examples of cataract which you have examined, you are able to understand readily nearly every case you may hereafter meet. The abnormal processes which produce cataracts are nearly identical in all cases; different varieties being but modifications of the same disease.

The differential diagnosis of the varieties of cataract is usually without difficulty, although, there may be occasional uncertainty regarding hard and soft cataract.

It is well to remember that before the age of thirty, cataracts are generally soft; in advanced age, hard. The presence of a central yellow-colored portion is often indicative of hard cataract. Soft cataracts are generally more rapid in their development than hard.

Nearly all hard cataracts are surrounded by soft cortical substance, resembling boiled starch.

Cataract may be complicated with amaurotic disease of the choroid, retina and optic nerve. Whenever the pupil is inactive under the influence of alternate light and shade, or dilates very slowly and imperfectly, from the use of atropia, and when a patient cannot see the light of a candle at night at the distance of fifteen feet, there is reason to fear that the removal of the cataract would not restore good vision.

Medical treatment of cataract is almost absolutely without benefit. Surgical interference is the only reliable means of relief. This usually consists in the use of the needle or of the knife. By the former instrument, the opaque lens is either depressed below the pupil behind the iris, or broken up and left in the eye to be absorbed. By the latter an incision is made in the cornea, through which the cataract is removed in substance.

For the discussion of the question whether to operate, when only one eye is affected, whether to operate upon both eyes at one time, and for the details of each mode of operating with the accidents, which are liable to occur, I must refer you to the common text books on diseases of the eye.

The following facts, however, I wish especially to impress upon you: that the passage of a large hard cataract through the pupil is liable to so bruise the iris as inevitably to produce iritis; this disease is often caused by fragments of the cortical substance resting in contact with the iris. To overcome these evils, it has been proposed, first to remove a portion of the iris as in iridectomy. If the extraction be performed some weeks after this preliminary operation, the lens not only passes through the pupil with less danger to the iris, but all the cortical substance is more readily removed. This mode of operating appears to be regarded with more and more favor by oculists in all cases of cataract in adults, whether hard or soft. In infancy there is less reason for iridectomy as a preparatory operation, since the iris is much less susceptible than in adults to the presence of portions of the lens lying in contact with it.

The disadvantages arising from the disfigured and enlarged pupils, occasioned by this mode of operating, are perhaps more than compensated by the increased chances of a favorable result.

I would urge upon you the careful perusal of all articles upon this subject you may meet.

